# Supplementary material for: Systematic analysis of paralogous regions in 41,755 exomes uncovers clinically relevant variation
Source: Nat Commun. 2023 Oct 27;14:6845. doi: 10.1038/s41467-023-42531-9 (PMC10611741; doi:10.1038/s41467-023-42531-9)
Supplement: Supplementary file 1 — Supplementary Information [file 41467_2023_42531_MOESM1_ESM.pdf]

Systematic analysis of paralogous regions in 41,755 exomes uncovers clinically relevant variation - Supplementary Information

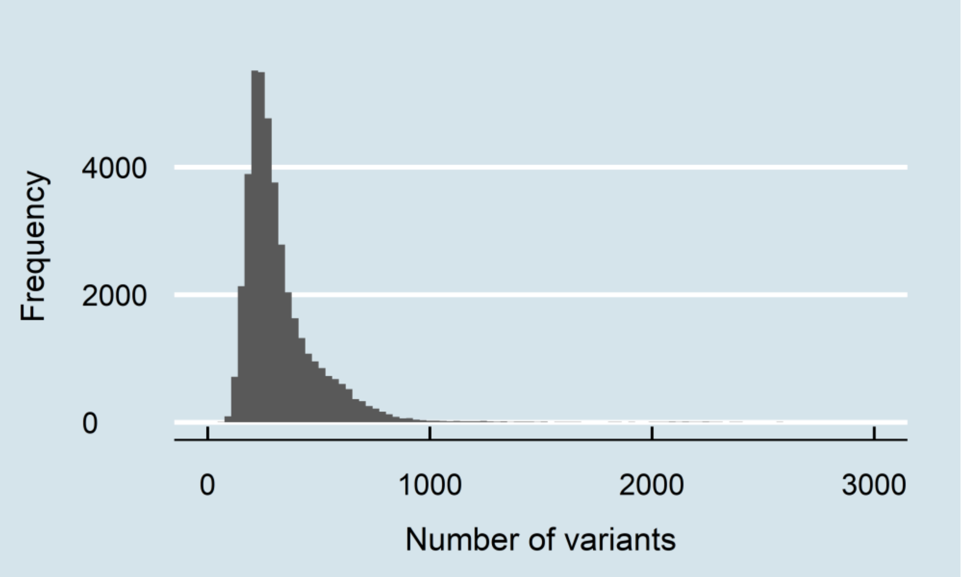

Supplementary Figure 1. Distribution of the number of SNVs/Indels (not the result of an ectopic gene conversion) per individual in the study cohort (n=41,755).

The horizontal axis indicates the number of SNVs/Indels (not the result of an ectopic gene conversion) that were identified per individual. The vertical axis represents the number of individuals with that number of variants identified. Clearly, the number of SNVs/Indels that were identified is between 0 and 1,000 for almost all individuals. Source data are provided as a Source Data file.

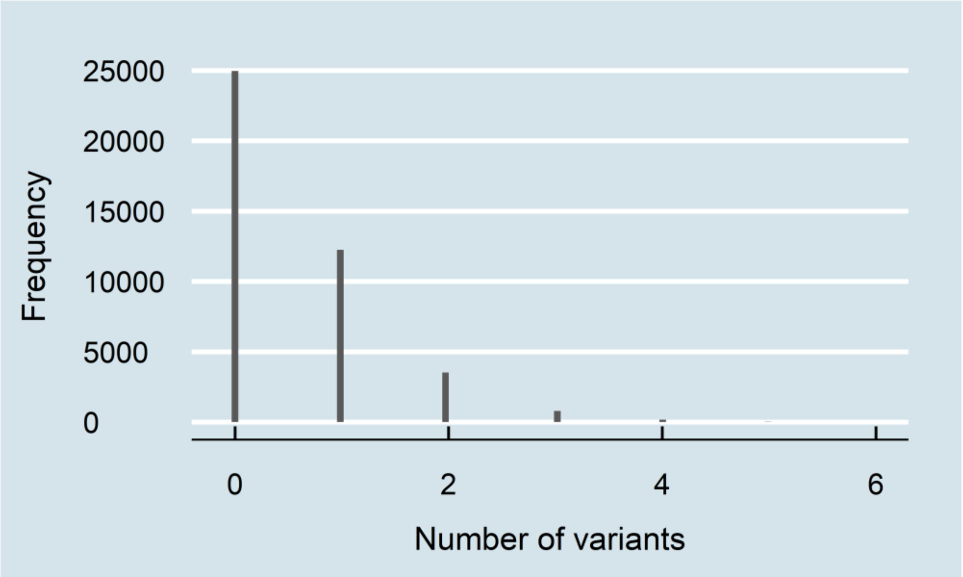

Supplementary Figure 2. Distribution of the number of homozygous gene conversions per individual in the study cohort (n=41,755).

The horizontal axis indicates the number of homozygous gene conversions that were identified per individual. The vertical axis represents the number of individuals with that number of gene conversions identified. In more than half of the studied individuals we did not identify a homozygous gene conversion. Source data are provided as a Source Data file.

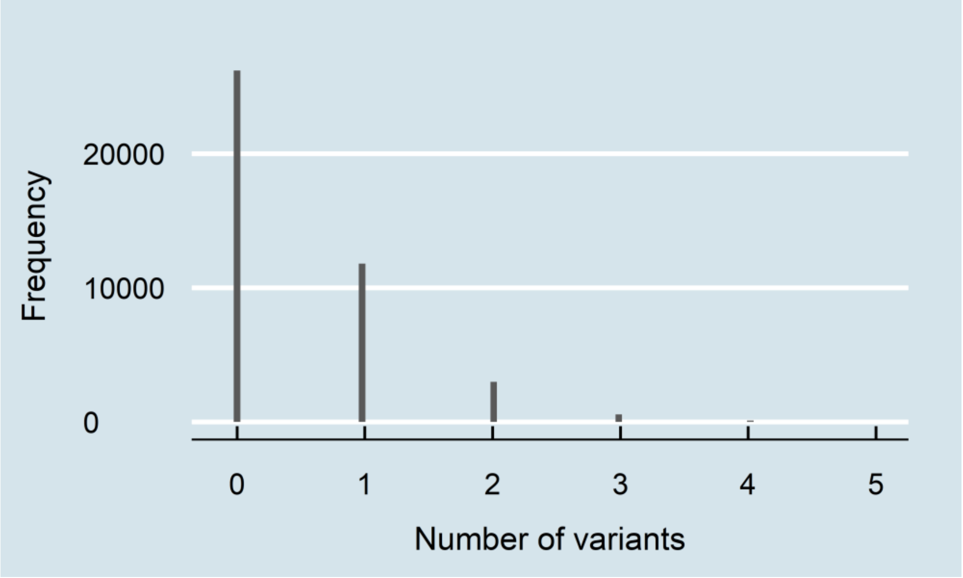

Supplementary Figure 3. Distribution of the number of homozygous deletions per individual in the study cohort (n=41,755).

The horizontal axis indicates the number of homozygous deletions that were identified per individual. The vertical axis represents the number of individuals with that number of gene conversions identified. In more than half of the studied individuals we did not identify a homozygous deletion. Source data are provided as a Source Data file.

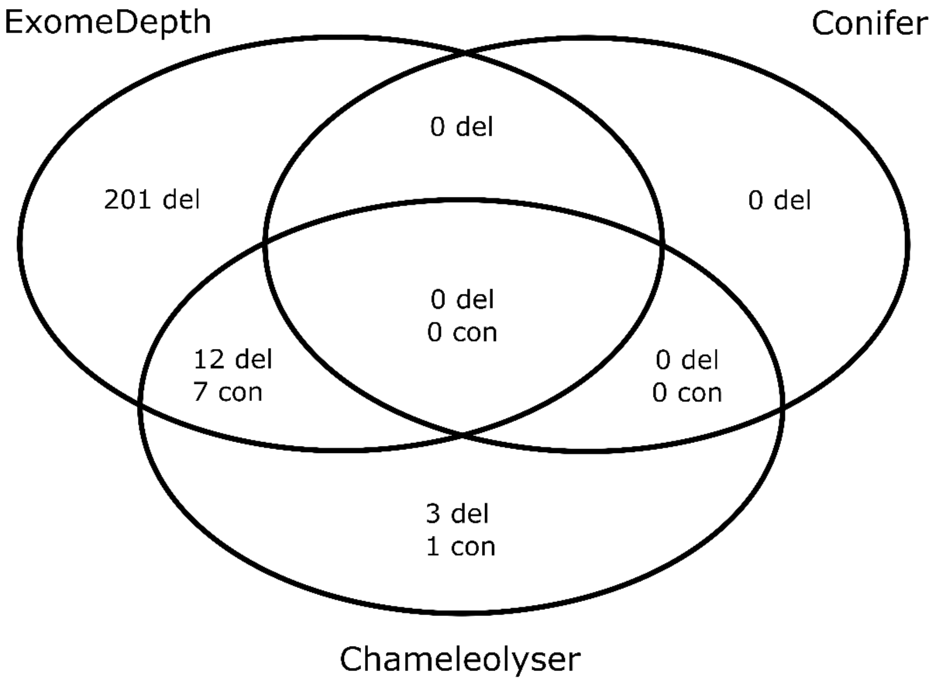

Supplementary Figure 4. Comparison between Chameleolyser, ExomeDepth and Conifer for the identification of homozygous deletions and gene conversions within the paralogous regions of 20 validation samples.

The Venn diagram shows the number of homozygous deletions (del) and the number of homozygous ectopic gene conversions (con) that are identified in the paralogous regions of the 20 exome samples for which LRS data was generated. The 7 conversions in the intersection between ExomeDepth and Chameleolyser are called as homozygous deletions by ExomeDepth.

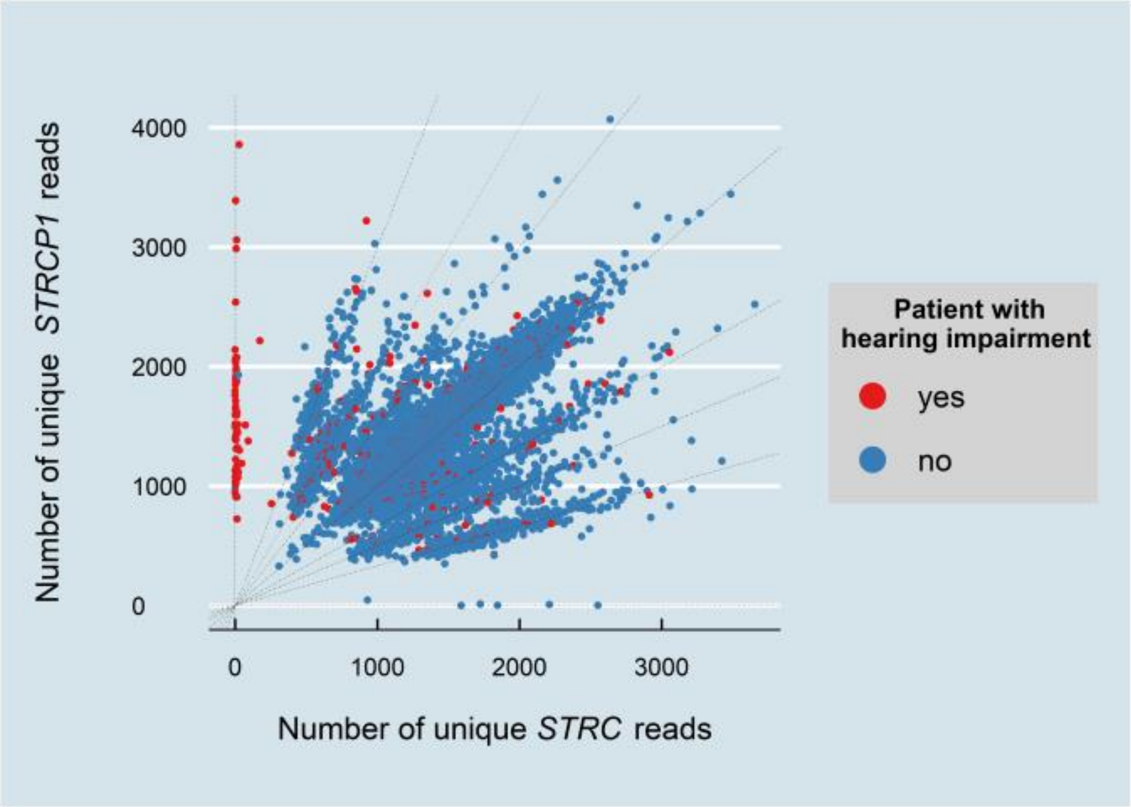

Supplementary Figure 5. The number of unique *STRC* versus *STRCP1* reads.

The horizontal axis represents the absolute number of uniquely aligned reads onto the last 6 exons of *STRC* (ENST00000450892). The vertical axis corresponds to the number of reads that uniquely align onto the homologous region of *STRCP1*. Each dot is an individual in the study cohort. The visualization is illustrative for the number of *STRC* and *STRCP1* copies each individual has. Patients with hearing impairment are coloured red. Point clouds are formed due to the (discrete) genetic nature of the events. Source data are provided as a Source Data file.

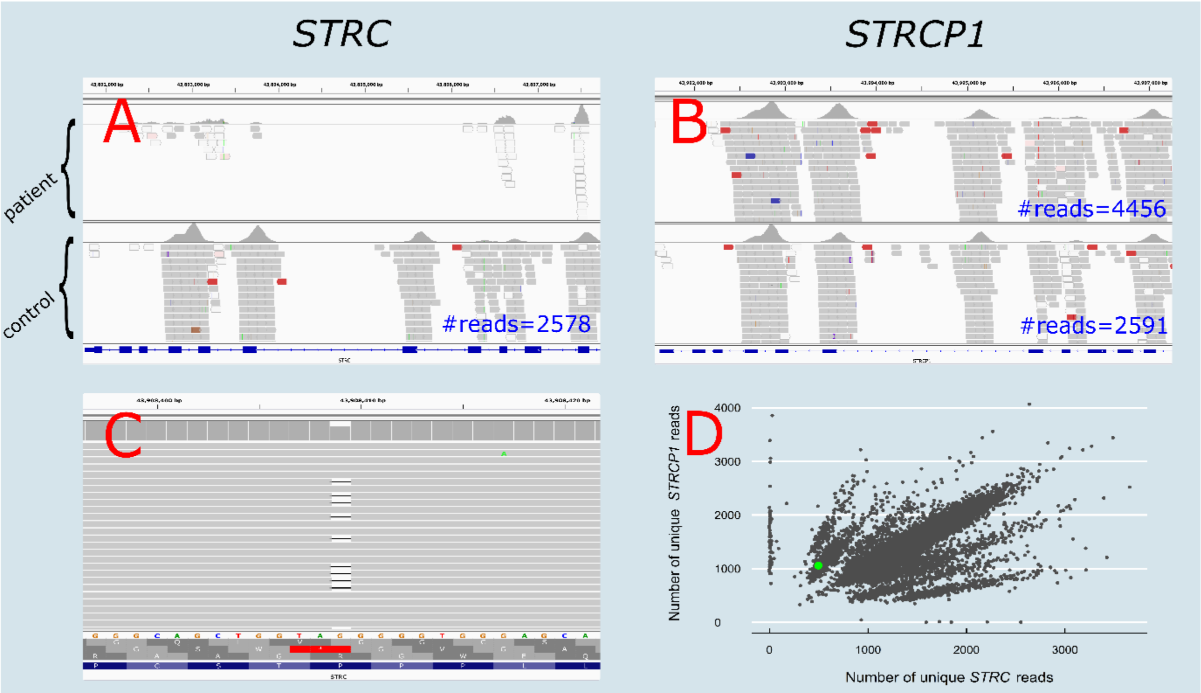

Supplementary Figure 6. Examples of pathogenic *STRC* variations identified by Chameleolyser.

Panels A and B illustrate a pathogenic homozygous ectopic gene conversion that was identified in *STRC*. Reads and a coverage track for both a patient with hearing impairment (SAMPLE_23606) and a control individual are displayed for the affected *STRC* exons (panel A) and for the homologous exons in *STRCP1* (panel B). The absolute number of reads that align to the reference sequence in the displayed window (shown in purple) is highly indicative for the presence of an ectopic conversion from *STRCP1* to *STRC* in the patient. Panels C and D illustrate the pathogenic variants in SAMPLE_38648. In panel C a single nucleotide deletion is shown in the masked alignment. Since reads from both *STRC* and *STRCP1* are aligned to the same locus, a heterozygous *STRC* variant has a variant allele fraction of roughly 0.25. Panel D is a replicate of supplementary figure 5, but here we coloured SAMPLE_38648 in green. Clearly, the patient is present in the point cloud corresponding to 1 *STRC* allele and 2 *STRCP1* alleles (a heterozygous *STRC* deletion).

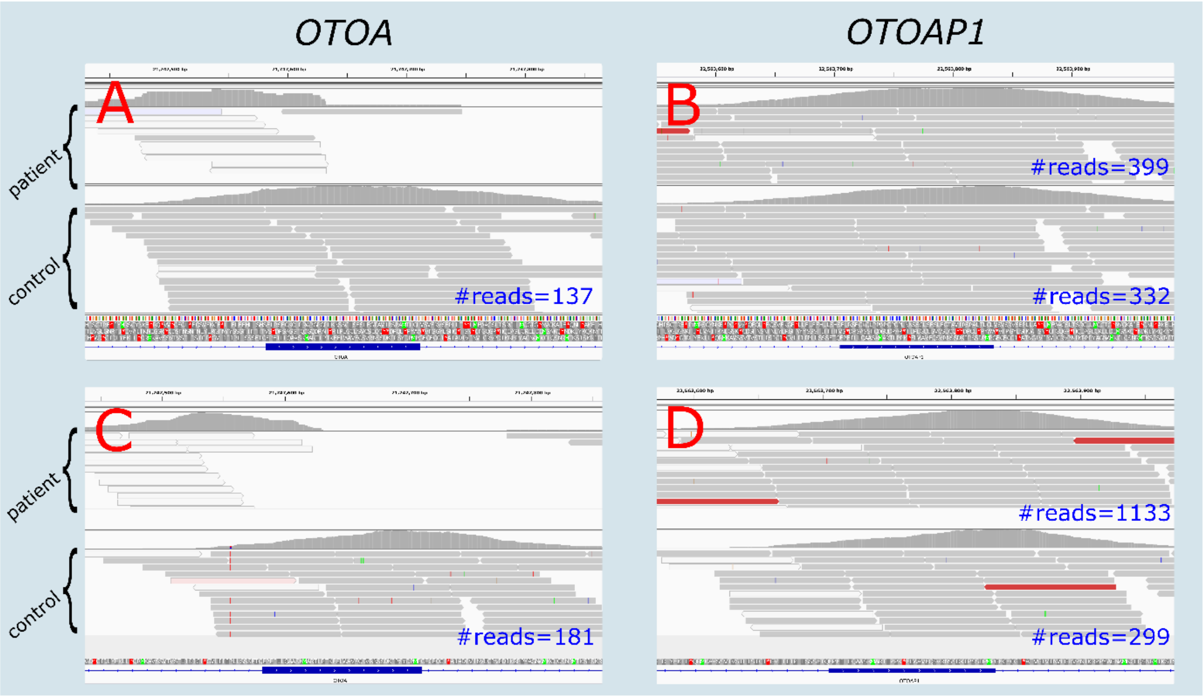

Supplementary Figure 7. Examples of pathogenic *OTOA* variations identified by Chameleolyser.

Panels A and B illustrate a pathogenic homozygous deletion that was identified in *OTOA*. Reads and a coverage track for both a patient with hearing impairment (SAMPLE_37062) and a control individual are displayed for the affected *OTOA* exon (panel A) and for the homologous exon in *OTOAP1* (panel B). The absolute number of reads that align to the reference sequence in the displayed window (shown in purple) is highly indicative for the presence of a homozygous deletion in the patient (no reads aligning onto the *OTOA* exon whereas the number of reads aligning onto the *OTOAP1* exon is comparable between the patient and the control). In panels C and D the same type of IGV screenshots are shown, but here the pathogenic event is a homozygous ectopic gene conversion which is strongly suggested by the absolute number of reads that are aligned onto *OTOAP1* in the patient (SAMPLE_24323).

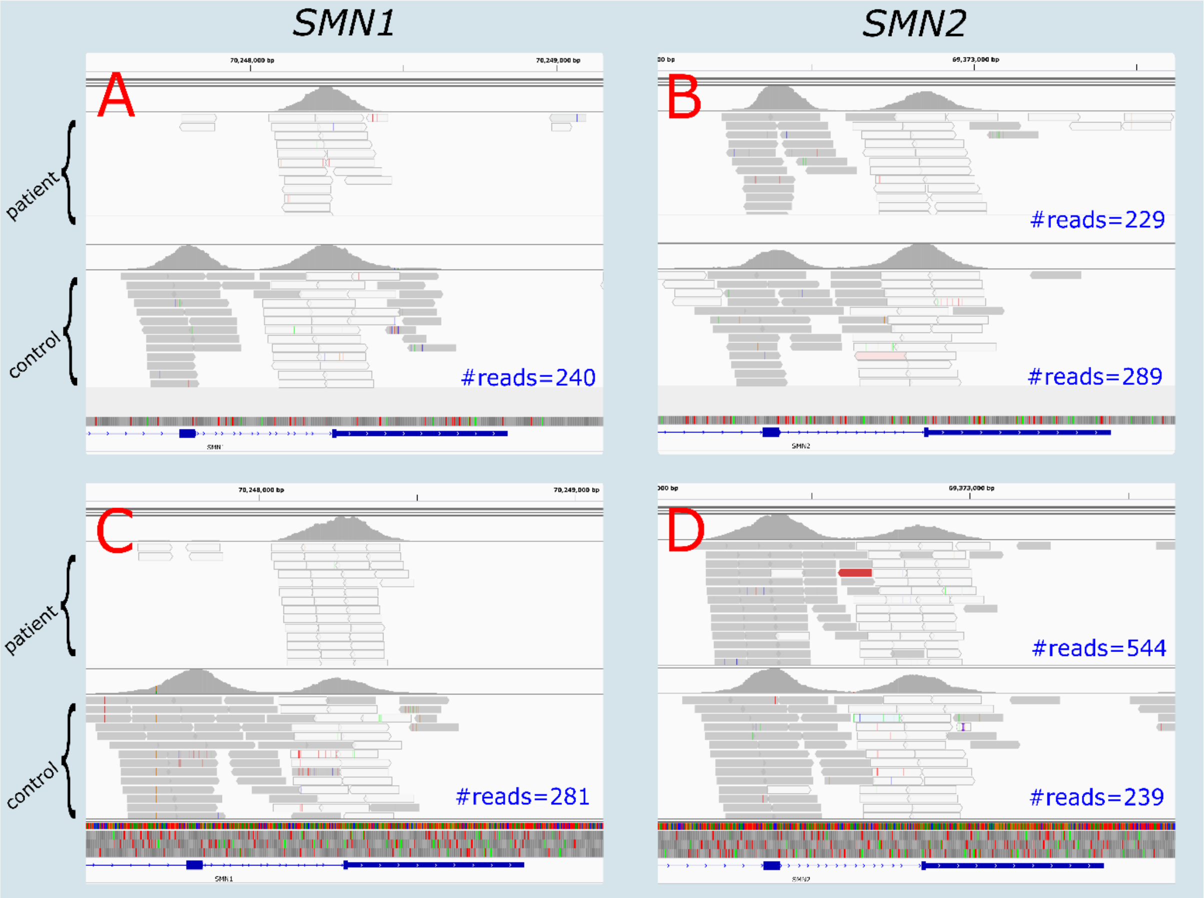

Supplementary Figure 8. Examples of pathogenic *SMN1* variations identified by Chameleolyser.

Panels A and B illustrate a pathogenic homozygous deletion that was identified in *SMN1*. Reads and a coverage track for both a patient with spinal muscular atrophy (SAMPLE_31987) and a control individual are displayed for the affected *SMN1* exons (panel A) and for the homologous exons in *SMN2* (panel B). The absolute number of reads that align to the reference sequence in the displayed window (shown in purple) is highly indicative for the presence of a homozygous deletion in the patient (no reads aligning onto the *SMN1* exon whereas the number of reads aligning onto the *SMN2* exon is comparable between the patient and the control). In panels C and D the same type of IGV screenshots are shown, but here the pathogenic event is a homozygous ectopic gene conversion which is strongly suggested by the absolute number of reads that are aligned onto SMN2 in the patient (SAMPLE_20848).

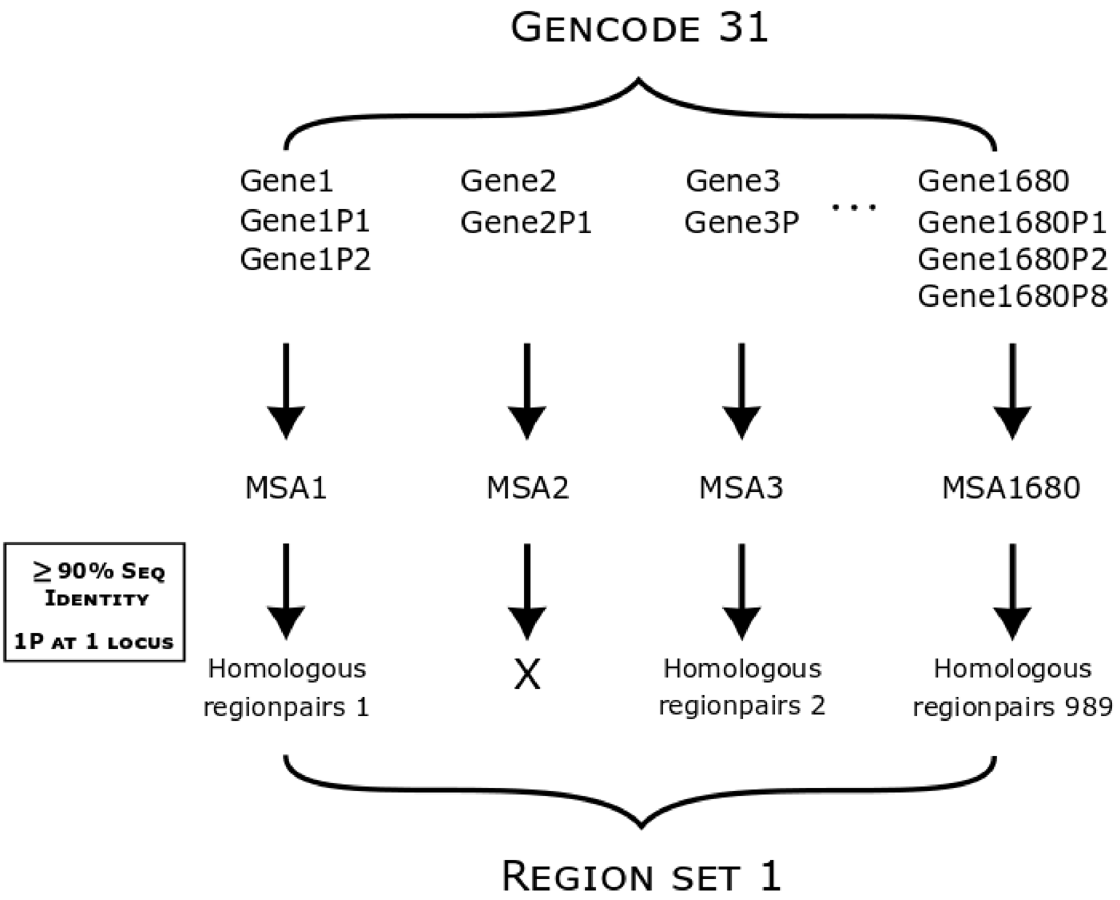

Supplementary Figure 9. Graphical summary of the derivation of region set I

This figure is a graphical representation of the derivation of region set I: regions in protein coding genes with known pseudogenes (Methods).

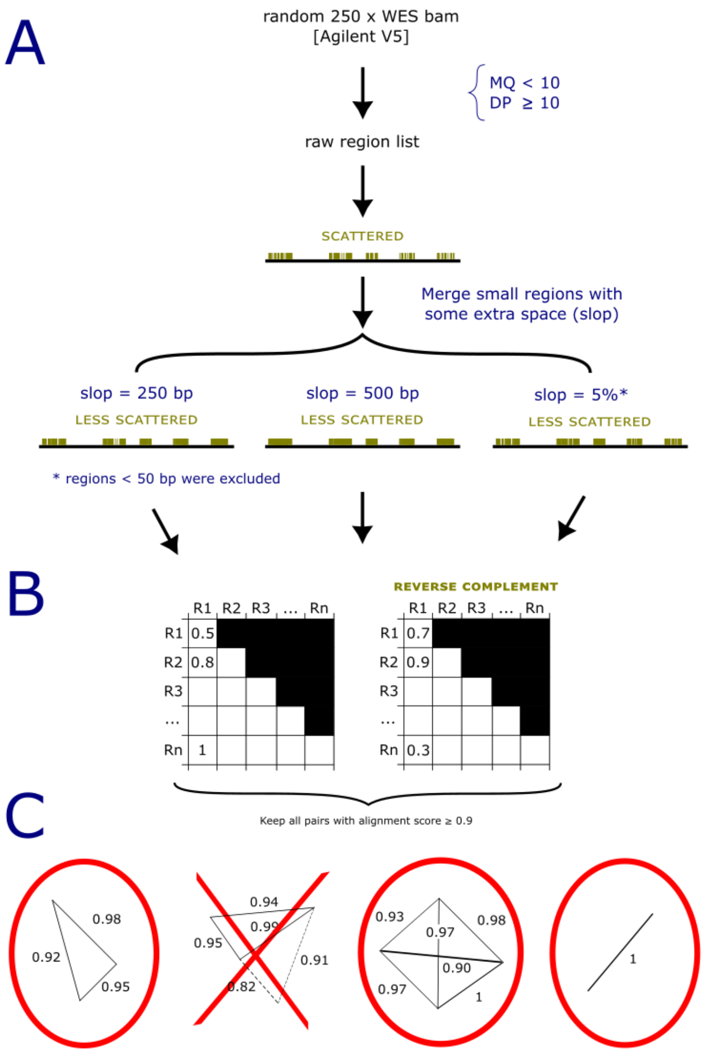

Supplementary Figure 10. Graphical summary of the derivation of region set II

This figure is a graphical representation of the derivation of region set II: regions that are associated with low mapping qualities (Methods). Panel A illustrates how low mapping quality regions were found in exome datasets. Panel B displays how these regions were pairwise aligned in order to identify sets of paralogous regions. Panel C shows how these initial sets of paralogous sequences were filtered to obtain reliable groups of paralogs.

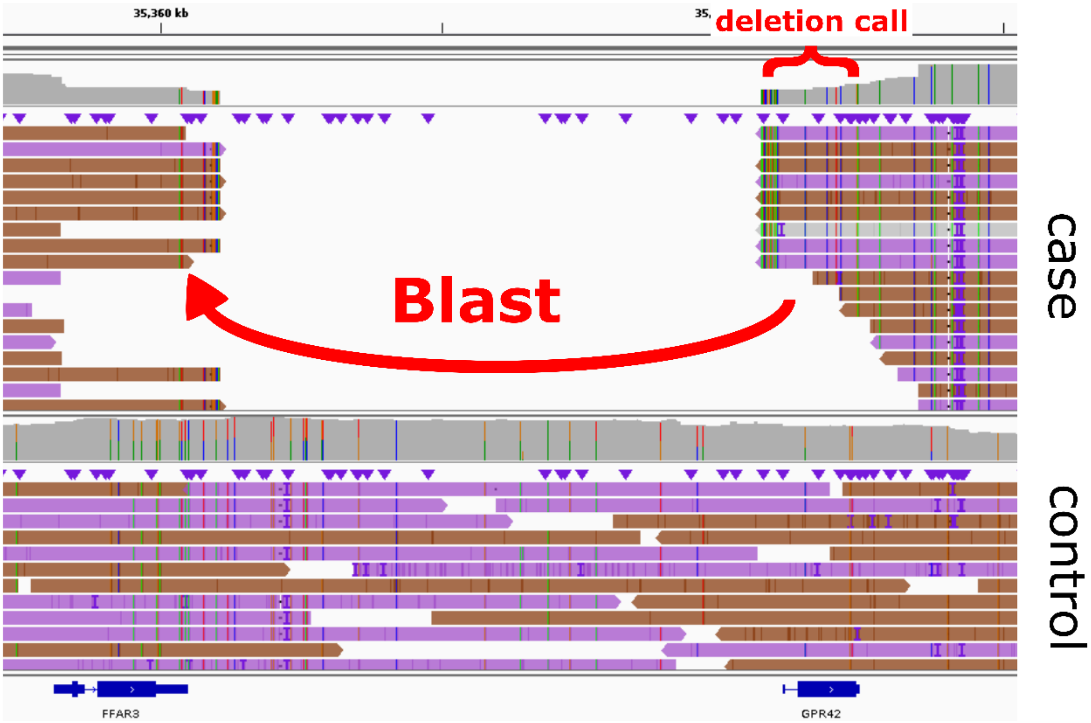

Supplementary Figure 11. Read alignment within deleted region

LRS alignments (and coverage track) are displayed for 2 individuals, a case and a control. In the case, as opposed to the control, there is a homozygous deletion. Chameleolyser called this homozygous deletion with the genomic region corresponding to the brace. The intergenic region between the 2 protein coding genes (*FFAR3* and *GPR42*) could not be included in the deletion call since Chameleolyser starts from WES data. Based on the LRS alignments, at first sight, the *GPR42* gene is not homozygously deleted since > 10 reads align onto it. However, the reads that align onto this gene have a large number of non-matching bases and the sequence perfectly corresponds to the *FFAR3* gene (*i.e.* without non-matching bases). Based on this we could conclude that the read alignment is suboptimal and that the region that Chameleolyser claims to be deleted is indeed deleted.

Supplementary Note 1. URLs for the genome-in-a-bottle data used in this study

HG002

- LRS: https://downloads.pacbcloud.com/public/revio/2022Q4/HG002-rep1/analysis/
- WES: https://ftp-trace.ncbi.nlm.nih.gov/ReferenceSamples/giab/data/AshkenazimTrio/HG002_NA24385_son/

OsloUniversityHospital_Exome/151002_7001448_0359_AC7F6GANXX_Sample_HG002-EEogPU_v02-KIT-Av5_AGATGTAC_L008.posiSrt.markDup.bam

HG003

- LRS: https://downloads.pacbcloud.com/public/revio/2022Q4/HG003-rep1/analysis/
- WES: https://ftp-trace.ncbi.nlm.nih.gov/ReferenceSamples/giab/data/AshkenazimTrio/HG003_NA24149_father/OsloUniversityHospital_Exome/151002_7001448_0359_AC7F6GANXX_Sample_HG003-EEogPU_v02-KIT-Av5_TCTTCACA_L008.posiSrt.markDup.bam

HG004:

- PacBio: https://downloads.pacbcloud.com/public/revio/2022Q4/HG004-rep1/analysis/
- WES: https://ftp-trace.ncbi.nlm.nih.gov/ReferenceSamples/giab/data/AshkenazimTrio/HG004_NA24143_mother/OsloUniversityHospital_Exome/151002_7001448_0359_AC7F6GANXX_Sample_HG004-EEogPU_v02-KIT-Av5_CCGAAGTA_L008.posiSrt.markDup.bam

HG005:

- LRS: https://ftp-trace.ncbi.nlm.nih.gov/ReferenceSamples/giab/data/ChineseTrio/analysis/PacBio_CCS_15kb_20kb_chemistry2_12072020/HG005/
- WES: https://ftp-trace.ncbi.nlm.nih.gov/ReferenceSamples/giab/data/ChineseTrio/HG005_NA24631_son/OsloUniversityHospital_Exome/151002_7001448_0359_AC7F6GANXX_Sample_HG005-EEogPU_v02-KIT-Av5_CGCATACA_L008.posiSrt.markDup.bam

NA12878/HG001

- LRS: https://ftp-trace.ncbi.nlm.nih.gov/ReferenceSamples/giab/data/NA12878/analysis/PacBio_CCS_15kb_20kb_chemistry2_042021/

- WES: https://ftp-trace.ncbi.nlm.nih.gov/ReferenceSamples/giab/data/NA12878/Nebraska_NA12878_HG001_TruSeq_Exome/NIST-hg001-7001-b-ready.bam

Supplementary Table 1. Sample identifier mapping between EGA and this manuscript for HiFi genome sequencing validation samples

Manuscript sample identifier	EGA sample identifier
SAMPLE_17545	EGAN00003613106
SAMPLE_17971	EGAN00003613113
SAMPLE_17973	EGAN00003613116
SAMPLE_17972	EGAN00003613117
SAMPLE_18244	EGAN00003613114
SAMPLE_18246	EGAN00003613115
SAMPLE_18245	EGAN00003613110
SAMPLE_18601	EGAN00003613111
SAMPLE_18603	EGAN00003613108
SAMPLE_18602	EGAN00003613109
SAMPLE_14580	EGAN00003613119
SAMPLE_14579	EGAN00003613121
SAMPLE_15442	EGAN00003613123
SAMPLE_15444	EGAN00003613105
SAMPLE_15443	EGAN00003613107
