# Supplementary material for: Systematic analysis of paralogous regions in 41,755 exomes uncovers clinically relevant variation
Source: Nat Commun. 2023 Oct 27;14:6845. doi: 10.1038/s41467-023-42531-9 (PMC10611741; doi:10.1038/s41467-023-42531-9)
Supplement: Supplementary file 3 — Description of Additional Supplementary Files [file 41467_2023_42531_MOESM3_ESM.pdf]

Systematic analysis of paralogous regions in 41,755 exomes uncovers clinically relevant variation - Description of Additional Supplementary Files

File Name: Supplementary Data 1

Description: Genomic coordinates of single nucleotide variant calls (not due to a gene conversion).

Column A (recurrence) represents the number of times the variant is observed in the study cohort. Column B shows the genomic coordinate of the variant (Ensembl default notation; hg19).

File Name: Supplementary Data 2

Description: Genomic coordinates of gene conversion calls.

Column A (recurrence) represents the number of times the event is observed in the study cohort. Column B, C and D respectively represent the chromosome, genomic start and genomic end of the event.

File Name: Supplementary Data 3

Description: Genomic coordinates of SNVs/Indels introduced by gene conversions.

Column A (recurrence) represents the number of times the variant is observed in the study cohort. Column B shows the genomic coordinate of the variant (Ensembl default notation; hg19).

File Name: Supplementary Data 4

Description: Genomic coordinates of deletion calls.

Column A (recurrence) represents the number of times the event is observed in the study cohort. Column B, C and D respectively represent the chromosome, genomic start and genomic end of the event.

File Name: Supplementary Data 5

Description: Overview of technical validation for SNVs/Indels (not due to a gene conversion) in 20 in-house validation samples.

Column A indicates in which sample the variant was identified. Column B displays the variant call. In column C the corresponding variant in the paralogous region can be found. Column D indicates whether or not the variant was found in the VCF file of the LRS data. If so, the variant call is validated (column E). If not, the variant was manually checked in the BAM file using IGV. Column F indicates whether or not the variant was clearly visible in the reads. If so, the variant call is validated (column G). Column H summarizes the total validation status (column E OR column G). Column I indicates whether or not the variant is rare (cohort allele frequency ≤ 0.5%).

File Name: Supplementary Data 6

Description: Overview of technical validation for gene conversions and deletions in in-house validation samples.

Column A indicates in which sample the variant was identified. Column B indicates whether the call is predicted to be a deletion or a gene conversion. Column F indicates whether or not the call is validated.

File Name: Supplementary Data 7

Description: Technical overview of SNVs/Indels (not due to a gene conversion) in the trios.

Column A shows the identifier for the particular child in each trio. There are 6,980 child-parent trios in total. Column B and D indicate the number of SNV/Indel calls in the particular proband (for 10% and 0.5% respectively). Column C and E indicate the number of variants that are not observed in the parents despite 60x read depth in the masked alignment (for 10% and 0.5% respectively; Methods). Overall, 99% of the variation that is observed in the child is inherited from one of the parents.

File Name: Supplementary Data 8

Description: Overview of technical validation for SNVs/Indels (not due to a gene conversion) in 5 genome-in-a-bottle samples.

Column A indicates in which sample the variant was identified. Column B displays the variant call. In column C the corresponding variant in the paralogous region can be found. Column D indicates whether or not the variant was found in the VCF file of the LRS data. If so, the variant call is validated (column E). If not, the variant was visually checked in the BAM file using IGV. Column F indicates whether or not the variant was clearly visible in the reads. If so, the variant call is validated (column G). Column H summarizes the total validation status (column E OR column G). Column I indicates whether or not the variant is rare (cohort allele frequency ≤ 0.5%).

File Name: Supplementary Data 9

Description: Comparison of the sensitivity for SNVs/Indels between Chameleolyser, GATK and DeepVariant.

Column A indicates the variant caller which is used. Cham_GATK refers to the union of the outputs from Chameleolyser and GATK. Sensitivity is calculated separately for variants residing in regions with zero mapping quality and variant residing in regions with strictly positive mapping qualities (column B). The number of true positive (TPs) and false negative (FNs) variants are displayed in columns C and D. The sensitivity is shown in column E.

File Name: Supplementary Data 10

Description: Comparison of homozygous deletion and gene conversion calls between Chameleolyser and ExomeDepth.

Column A indicates the sample in which the event was called. Column B displays the coordinates of the deletion call for ExomeDepth. If Chameleolyser calls an overlapping deletion or gene conversion, it is shown in column C. Column D indicates the hg38 coordinates of the variant call. When there is no 1:1 map between hg19 and hg38, the value ‘NA’ is used. The last column shows the number of HiFi reads in the region.

File Name: Supplementary Data 11

Description: Variants with ambiguous positions.

Every variant call in the table either corresponds to a missense or LoF-variant (Genomic coordinate in column B; Ensembl consequence in column C; Canonical transcript in column D), or to a variant in the non-coding space of the genome (Genomic coordinate in column F; Ensembl consequence in column G; Canonical transcript in column H). LRS data allows us to evaluate which one of these 2 possible variants is the actual genomic alteration causing the variant call by Chameleolyser (column E for the coding and column I for the non-coding space).

File Name: Supplementary Data 12

Description: Number of synonymous, missense and LoF variants identified by GATK in the full exome.

Column A shows the identifier for the individual in the study cohort. Column B, C and D respectively represent the number of synonymous, missense and LoF variants in the exome.

File Name: Supplementary Data 13

Description: Number of synonymous, missense and LoF VAPs identified by Chameleolyser.

Column A shows the identifier for the individual in the study cohort. Column B, C and D respectively represent the number of synonymous, missense and LoF variants in the paralogous regions of the exome as determined by Chameleolyser.

File Name: Supplementary Data 14

Description: Overview of the cohort of 41,755 WES samples.

Column A shows the identifier for the individual in the study cohort. Column B represents an identifier for the family. Column C indicates which member of the family the sample is (Child, father, mother or single sample). In column D the enrichment kit can be found. The type of sequencing machine that is used to generate the underlying data can be found in column E. Read length of the respective sequencing reads are displayed in column F. The molecular status before we conducted our analysis is displayed in column G.

File Name: Supplementary Data 15

Description: SNVs/Indels (not due to gene conversions) of clinical interest.

Column A indicates in which sample the variant was identified. Column B corresponds to the variant call. Column C is the other possible genetic variant that could give rise to the variant call in column B (VAP). The gene symbol is displayed in column D. The Ensembl consequence of the variant, relative to the canonical transcript (column F) is represented in column E. The variant allele fraction of the variant in the masked alignment is indicated in column G. The number of times that the variant is observed in the study cohort can be found in column H. Whether or not the variant is wet-lab tested is depicted in column I. In case the variant is confirmed as an alteration of the protein coding gene, a ‘1’ is displayed in the very last column.

File Name: Supplementary Data 16

Description: Homozygous ectopic gene conversions of clinical interest.

Column A indicates in which sample the variant was identified. Column B, C and D respectively represent the chromosome, genomic start and genomic end of the ectopic gene conversion event. The number of times the event is observed in our study cohort can be seen in column E. The variant that is introduced by means of the gene conversion can be found in column F. Gene symbol and the Ensembl consequence of the variant (relative to the canonical transcript) can respectively be found in columns G and H.

File Name: Supplementary Data 17

Description: *STRC* and *STRCP1* coverage for the study cohort.

Data table corresponding to supplementary figure 4. Column A represents the sample. Column B represents the number of reads that uniquely align onto the last 6 exons of *STRC*. The number of reads that align uniquely onto the paralogous sequence of *STRCP1* are displayed in column C. The ratio (column B divided by column C) can be found in column D. The percentage of all reads that originate from *STRC* can be found in column E (B/(B+C)). Based on this, the number of *STRC* and *STRCP1* copies can be estimated (the estimate corresponds to the closest theoretical ratio, see supplementary figure 4). In case the deafness disease gene panel was requested, a ‘1’ is displayed in column H.

File Name: Supplementary Data 18

Description: Homozygous deletions of clinical interest.

Column A indicates in which sample the deletion was identified. Column B, C and D respectively represent the chromosome, genomic start and genomic end of the event. The number of times the event is observed in our study cohort can be seen in column E. The gene symbol of the gene that is affected by the deletion can be found in column F.

File Name: Supplementary Data 19

Description: Homozygous *STRC* deletions and gene conversions in patients with hearing impairment.

Amongst the group of patients with hearing impairment, 58 homozygous *STRC* deletions were identified with WES. MLPA confirmed these events (column F). With Cameleolyser, 22 of these events are predicted to be gene conversions instead of deletions (column E). Respective sample and genomic coordinates can be found in columns A-D.

File Name: Supplementary Data 20

Description: Homozygous conversions from STRCP1 to STRC that do not introduce LoF variants into STRC

In our cohort of 41,755 we identified 47 ectopic gene conversions from *STRCP1* to *STRC* that do not introduce LoF variants into STRC. Column A represents the identifier of the individual. Columns B, C and D correspond to respectively the chromosome, genomic start and genomic end of each event. For clarity it is also indicated that all of these events are called as homozygous gene conversion events with Chameleolyser (column E) and homozygous deletions with Exome Depth (column F).

File Name: Supplementary Data 21

Description: Overview of all genes implicated in the homology analysis.

Column A represents the gene symbol of the gene that is affected by sequence homology and therefore incorporated in our homology study. In column B the type of the gene product can be found (Ensembl 97). Ensembl stable gene id and stable canonical transcript id can respectively be found in columns C and D. If coding exons of the canonical transcript are affected, they are listed in column E.

Column F represents the ratio of exons that are involved in the analyses and the total number of exons in the canonical transcript. The total number of coding exons, over all transcripts, is displayed in column G. The number of coding bases can be found in column H. The phenotype that is linked to the respective gene is shown in the last column.

File Name: Supplementary Data 22

Description: Genomic alignment between *CELA3A* (chr1:22338346-22339613) and *CELA3B* (chr1:22315158-22316429).

An ectopic gene conversion from *CELA3B* to *CELA3A* introduces variants into *CELA3A*, i.e. the sequence differences between both genes.

File Name: Supplementary Data 23

Description: List of variant calling regions.

Column A, B and C respectively represent the chromosome, start and end of the variant calling regions in this study.

File Name: Supplementary Data 24

Description: List of regions for read extraction.

Column A, B and C respectively represent the chromosome, start and end of the read extraction regions in this study.

File Name: Supplementary Data 25

Description: List of regions to be masked in re-alignment of extracted reads.

Column A, B and C respectively represent the chromosome, start and end of the regions that are masked in the reference sequence in the re-alignment of the extracted reads.

File Name: Supplementary Data 26

Description: Overview of subregions for deletion and gene conversion calling.

Regions in which deletion and gene conversion identification was conducted. All regions come in pairs: exactly 2 regions that are homologous.
