# Supplementary material for: Systematic analysis of paralogous regions in 41,755 exomes uncovers clinically relevant variation
Source: Nat Commun. 2023 Oct 27;14:6845. doi: 10.1038/s41467-023-42531-9 (PMC10611741; doi:10.1038/s41467-023-42531-9)
Supplement: Supplementary file 30 — Reporting Summary [file 41467_2023_42531_MOESM30_ESM.pdf]

Corresponding author(s): Christian Gilissen

Last updated by author(s): Aug 23, 2023

Reporting Summary

Nature Portfolio wishes to improve the reproducibility of the work that we publish. This form provides structure for consistency and transparency in reporting. For further information on Nature Portfolio policies, see our Editorial Policies and the Editorial Policy Checklist.

Statistics

For all statistical analyses, confirm that the following items are present in the figure legend, table legend, main text, or Methods section.

n/a	Confirmed	
☐	☒	The exact sample size (*n*) for each experimental group/condition, given as a discrete number and unit of measurement
☒	☐	A statement on whether measurements were taken from distinct samples or whether the same sample was measured repeatedly
☒	☐	The statistical test(s) used AND whether they are one- or two-sided<br>*Only common tests should be described solely by name; describe more complex techniques in the Methods section.*
☒	☐	A description of all covariates tested
☒	☐	A description of any assumptions or corrections, such as tests of normality and adjustment for multiple comparisons
☒	☐	A full description of the statistical parameters including central tendency (e.g. means) or other basic estimates (e.g. regression coefficient) AND variation (e.g. standard deviation) or associated estimates of uncertainty (e.g. confidence intervals)
☒	☐	For null hypothesis testing, the test statistic (e.g. F, t, r) with confidence intervals, effect sizes, degrees of freedom and P value noted<br>*Give P values as exact values whenever suitable.*
☒	☐	For Bayesian analysis, information on the choice of priors and Markov chain Monte Carlo settings
☒	☐	For hierarchical and complex designs, identification of the appropriate level for tests and full reporting of outcomes
☒	☐	Estimates of effect sizes (e.g. Cohen's *d*, Pearson's *r*), indicating how they were calculated

Our web collection on statistics for biologists contains articles on many of the points above.

Software and code

Policy information about availability of computer code

Data collection	NA
Data analysis	Tools that were only used to generate the list of paralogous regions include: mafft 7.407 and EMBOSS Needle v 6.6.0.0. Other tools used in this work are listed in the environment of the Chameleolyser tool which is available on GitHub (https://github.com/Genome-Bioinformatics-RadboudUMC/Chameleolyser/).

For manuscripts utilizing custom algorithms or software that are central to the research but not yet described in published literature, software must be made available to editors and reviewers. We strongly encourage code deposition in a community repository (e.g. GitHub). See the Nature Portfolio guidelines for submitting code & software for further information.

Data

Policy information about availability of data

All manuscripts must include a data availability statement. This statement should provide the following information, where applicable:

- Accession codes, unique identifiers, or web links for publicly available datasets
- A description of any restrictions on data availability
- For clinical datasets or third party data, please ensure that the statement adheres to our policy

The exome datasets onto which the method is applied are part of the routine genetic investigation from Genome Diagnostics Nijmegen and cannot be shared due to data privacy laws. The data that support the findings of this study are available as Supplementary data 1–26. The genome-in-a-bottle data used in this study are publicly on NCBI (URLs available on GitHub (https://github.com/genome-in-a-bottle/giab_data_indexes)) and/or the PacBio cloud (https://

downloads.pacbcloud.com/public/). A list of download URLs per sample is also available as Supplementary Note 1.LRS data that was used for validation purposes is available under accession numbers EGAS00001006479 (long-read genome sequencing for individuals with biobank consent) and EGAS00001007513 (STRC amplicon sequencing). These datasets are available under restricted access. Re-use of the data will be evaluated by a data access committee to evaluate whether the proposed re-use is in line with the consent. Supplementary table 1 describes the mapping between the EGA sample identifiers and the identifiers that were used in this manuscript. Source data are provided with this paper.

Research involving human participants, their data, or biological material

Policy information about studies with human participants or human data. See also policy information about sex, gender (identity/presentation), and sexual orientation and race, ethnicity and racism.

Reporting on sex and gender	With our method we want to identify genetic events in duplicated genomic regions, independent from sex. In general, we did not differentiate between males and females in this work. Gender is not relevant for genetic rare diseases.
Reporting on race, ethnicity, or other socially relevant groupings	We include data from all individuals for which exome sequencing was requested between 2002 and 2020 as part of the routine genetic investigation from Genome Diagnostics Nijmegen. We did not exclude anyone based on sex, gender, ethnicity, race, age or any other socially relevant groupings.
Population characteristics	The study cohort 41,755 individuals consists of 17,650 previously genetically undiagnosed rare disease patients.
Recruitment	We include data from all individuals for which exome sequencing was requested between 2002 and 2020 as part of the routine genetic investigation from Genome Diagnostics Nijmegen.
Ethics oversight	A diagnostic laboratory can use (de-identified) samples from archived clinical samples to validate and implement novel diagnostic assays. The derived clinically relevant variants can be shared, but in absence of explicit data sharing consent at individual patient level, FASTQ, BAM and VCFs cannot be disclosed. These methods are also in accordance with relevant guidelines and regulations and approved by the institutional review board of the Radboud University Medical Center (2020-7142) and the Declaration of Helsinki.

Note that full information on the approval of the study protocol must also be provided in the manuscript.

Field-specific reporting

Please select the one below that is the best fit for your research. If you are not sure, read the appropriate sections before making your selection.

☒ Life sciences ☐ Behavioural & social sciences ☐ Ecological, evolutionary & environmental sciences

For a reference copy of the document with all sections, see nature.com/documents/nr-reporting-summary-flat.pdf

Life sciences study design

All studies must disclose on these points even when the disclosure is negative.

Sample size	Sample size (n=41,755) corresponds to the number of diagnostic exomes that were sequenced between 2002 and 2020 in Genome Diagnostics Nijmegen. Our method does not require a specific sample size because it can be applied on a single-sample basis. Nevertheless, applying the method on a larger cohort will result in more genetic diagnosis. Also, it enabled us to filter for rare variants which is highly relevant for rare disease research and diagnostics.
Data exclusions	We did not exclude any data.
Replication	Our study design does not require replication since we do not measure any variable which is dependent on multiple factors. We uncover genetic variants which are present in sequencing data and validate these results.
Randomization	Our study design does not require randomisation since we do not measure any variable which is dependent on multiple factors. We uncover genetic variants which are present in sequencing data and validate these results.
Blinding	Our study design does not require blinding. We did not allocate individuals to a specific group.

Reporting for specific materials, systems and methods

We require information from authors about some types of materials, experimental systems and methods used in many studies. Here, indicate whether each material, system or method listed is relevant to your study. If you are not sure if a list item applies to your research, read the appropriate section before selecting a response.

Materials & experimental systems

n/a	Involved in the study	
☒	☐	Antibodies
☒	☐	Eukaryotic cell lines
☒	☐	Palaeontology and archaeology
☒	☐	Animals and other organisms
☒	☐	Clinical data
☒	☐	Dual use research of concern
☒	☐	Plants

Methods

n/a	Involved in the study	
☒	☐	ChIP-seq
☒	☐	Flow cytometry
☒	☐	MRI-based neuroimaging
